# Sirtuin 2 Regulates Microvascular Inflammation during Sepsis

**DOI:** 10.1155/2017/2648946

**Published:** 2017-04-19

**Authors:** Nancy Buechler, Xianfeng Wang, Barbara K. Yoza, Charles E. McCall, Vidula Vachharajani

**Affiliations:** ^1^Department of Anesthesiology, Wake Forest School of Medicine, Winston-Salem, NC, USA; ^2^Department of Surgery, Wake Forest School of Medicine, Winston-Salem, NC, USA; ^3^Department of Internal Medicine, Wake Forest School of Medicine, Winston-Salem, NC, USA

## Abstract

*Objective*. Sepsis and septic shock, the leading causes of death in noncoronary intensive care units, kill more than 200,000/year in the US alone. Circulating cell-endothelial cell interactions are the rate determining factor in sepsis inflammation. Sirtuin, a seven-member family of proteins (SIRT1–7), epigenetically controls inflammation. We have studied the roles of SIRTs 1, 3, and 6 in sepsis previously. In this project, we studied the role of SIRT2 on sepsis-related inflammation. *Methods*. Sepsis was induced in C57Bl/6 (WT), SIRT2 knockout (SIRT2KO), and SIRT2 overexpressing (SIRT2KI) mice by cecal ligation and puncture (CLP). We studied leukocyte/platelet adhesion using intravital microscopy and E-selectin/ICAM-1 adhesion molecule expression in the small intestine with immunohistochemistry (IHC) six hours post-CLP/sham surgery. We also studied 7-day survival rates in WT, SIRT2KO, and SIRT2KI sepsis mice. *Results*. Compared to WT mice, SIRT2KO mice show exaggeration while SIRT2KI mice show attenuation of cellular adhesion with sepsis in the small intestine. We also show that the small intestinal E-selectin and ICAM-1 expressions increased in SIRT2KO and decreased in SIRT2KI mice versus those in WT sepsis mice. We show that the 7-day survival rate is decreased in SIRT2KO and increased in SIRT2KI sepsis mice. *Conclusion*. SIRT2 modulates microvascular inflammation in sepsis and affects survival.

## 1. Introduction

Sepsis and septic shock, the most expensive conditions in the US, are the leading causes of death in the noncoronary intensive care units [1, 2]. More than 30 different clinical trials for sepsis failed, leaving no molecular-based therapies currently available [3]. Sepsis leads to multiple organ failures; the number of organs involved correlates with mortality [4]. In a dysregulated acute systemic inflammatory response of sepsis, microvasculature sits at an interface between systemic circulation and local tissue. Circulating cell-endothelial cell interactions at the microvascular interface (MVI) are the rate-limiting factors in inflammation [5]. We and others have studied the role of microvascular response in early sepsis previously [6, 7]. Knowing how the MVI-cell responses are directed is critical to designing molecular-based treatments.

The sirtuin family of proteins is among the many regulators of cellular homeostasis [8, 9]. Sirtuins, first described in yeast, are the highly conserved family of proteins. The seven mammalian sirtuins (SIRT1–7) disperse through different cell compartments to guard homeostasis, sense bioenergy needs of cell, and make changes to nutrient sources [10]. SIRT1 and 6 are nuclear, SIRT7 nucleolar, SIRT3, 4, and 5 mitochondrial, and SIRT2 is cytoplasmic under basal homeostasis conditions [11]. Nuclear SIRT1, the most extensively studied sirtuin, epigenetically directs the pro- versus anti-inflammatory response in sepsis [12]. We reported that nuclear SIRT1and SIRT6 and mitochondrial SIRT3 inform immune and metabolic (immunometabolic) programming during acute inflammation and are dysregulated during sepsis [13, 14].

SIRT2, originally described as a tubulin deacetylase, modifies acetylation of many histone and nonhistone proteins including transcription factors [15]. While described as a cytoplasmic protein, SIRT2 translocates to nucleus during mitosis and cellular stress [16]. Nucleocytoplasmic shuttling of SIRT2 likely regulates its biological activity [17]. Mounting evidence suggests a critical role for SIRT2 in the control of inflammation, immunometabolism, and tumorigenesis [15]. We have shown recently that SIRT2, the most abundant of sirtuins in the adipose tissue, also regulates hypoinflammatory response of late sepsis in obese mice with sepsis via direct deacetylation of NFĸB p65 [18] consistent with literature implicating the role of SIRT2 in neuroinflammation and collagen-induced arthritis among other inflammatory conditions [19, 20].

The effect of SIRT2 modulation on microvascular inflammation during early sepsis is not very well studied. Here, we report a role for SIRT2 in inflammation during early sepsis. Using a genetic approach to modulate SIRT2 in mice, we show that in early sepsis, low SIRT2 levels enhance and high SIRT2 levels attenuate the MVI inflammatory response in vivo via modulation of adhesion molecule expression impact sepsis mortality rates in rodent sepsis.

## 2. Material and Methods

### 2.1. Mice

The studies were approved by the Institutional Animal Care and Use Committee of the Wake Forest School of Medicine; experiments were performed according to the NIH guidelines. The wild type (WT: C57Bl/6) mice were purchased from Jackson Laboratories; SIRT2 knockout (*Sirt2^tm1.1Fwa;^* SIRT2KO) and SIRT2 overexpressing (SIRT2 knock in SIRT2KI) were bred in the Wake Forest Animal facility. The SIRT2KI and SIRT2KO mice were on C57Bl/6 background.

The WT mice and SIRT2KO breeding pairs were obtained from Jackson Laboratories (Bar Harbor, ME, USA), and the SIRT2KI breeding pair was a generous gift from Dr. Sinclair (Harvard Medical School, Boston, MA) and Dr. Lindsay Wu (University of New South Wales, Australia). These mice have been used in literature before [21]. The method for creation of these mice is described in literature. Briefly, Cre-inducible SIRT2 transgenic mice were created using transcriptional STOP element flanked with loxP sites which was then inserted between a CAGGS promoter and the murine SIRT2 cDNA. The construct was then targeted into the mouse collagen A1 locus using flp recombinase-mediated genomic integration into C10 ES cells (derived from V6.5; BL/6 × 129Sv/Jae F1 ES cells). Mouse embryonic stem cells carrying a single copy of the SIRT2STOP construct were identified using resistance to the antibiotic marker hygromycin and Southern blotting, clones injected into blastocysts and pups genotyped for positive recombination. Mice were then backcrossed to C57BL/6 to 10 generations. Subsequently, SIRT2STOP mice were crossed with CMV-Cre transgenic mice strains on C57BL/6J background (Jackson Labs, Bar Harbor, ME, USA) to generate constitutive SIRT2 transgenic animals (SIRT2tg). SIRT2tg; CMV-Cre double transgenics were then backcrossed to C57BL/6J to outcross the CMV-Cre allele [21]. Six eight-week old mice were used for experiments.

SIRT2KO mice breeding pair was purchased from Jackson laboratory. Per vendor description, a targeting vector was designed to replace exon 5-6 part and part of exon 7 of the mouse SIRT2 gene with a floxed neomycin-resistance cassette. This construct was then electroporated into 129S6/SvEvTac-derived TC1 embryonic stem cells. Correctly targeted embryonic stem cells were then transiently transfected with a Cre expression plasmid for the purpose of removing neomycin cassette. Embryonic stem cells that had successfully undergone Cre-mediated recombination and no longer retained the cassette were injected into C57BL/6J blastocytes, and the resulting males were bred to 129/Sv females. The offsprings were then backcrossed to C57Bl/6 for 8 generations to produce homozygous SIRT2KO mice. After arrival to Jackson Laboratories, these mice were then bred for at least one generation to establish colony (https://www.jax.org/strain/012772).

### 2.2. Cecal Ligation and Puncture (CLP)

Anesthetized mice (using 1–3% Isoflurane/O2 mixture via nose cone) underwent laparotomy. A one-centimeter laparotomy incision was performed, cecum identified, ligated, perforated two times with a 22-guage needle and abdomen closed in two layers (peritoneum and skin) as described in literature [22]. Each mouse received subcutaneous fluid resuscitation of 1 ml normal saline. Sham-operated mice underwent laparotomy followed by fluid resuscitation, without cecal ligation or puncture.

### 2.3. Intravital Fluorescent Video Microscopy (IVM)

Anesthetized mice (ketamine 150 mg/kg + xylazine 7.5 mg/kg; intramuscularly) underwent carotid artery and jugular vein cannulations followed by intravital microscopy procedures 6 h post-CLP as described previously [22]. The laparotomy incision (from previous CLP/sham surgery) was opened to exteriorize the small intestine to study microcirculation (*n* = 4–6 mice/group) as described previously [22]. Rhodamine 6G (Sigma-Aldrich, St. Louis, MO, USA; 0.02% in 100 *μ*l PBS) was injected intravenously to visualize leukocytes (labeled red). Platelets from matched donors were labeled green ex vivo using carboxyfluorescein diacetate succinimidyl ester (CFSE: Sigma-Aldrich; St. Louis, MO, USA; 90 *μ*M). This allowed leukocyte and platelet visualization simultaneously.

Platelets were obtained from donor mice of the same genetic background. Platelet donor mice underwent carotid arterial cannulation under ketamine+xylazine anesthesia (ketamine 150 mg/kg + xylazine 7.5 mg/kg; intramuscularly) to obtain blood; platelets were isolated and labeled with CFSE as described previously [7, 23]. Labeled platelets (*n* = 100 × 10^6^) were infused via jugular venous cannula over 5 min (yielding <5% of the total platelet count) and allowed to circulate for additional 5 min before recording on a DVD. Evidence suggests that these platelet isolation/labeling procedures do not affect activity/viability of isolated platelets [24].

Three to five postcapillary venules per mouse were recorded (4–6 mice per group) (1 min 20 seconds each venule) and cell adhesion quantified from one minute of the recording. A cell (leukocyte/platelet), stationary for at least 30 seconds (consecutive) of the one-minute recording analyzation, was considered adherent. The mean of the average values of leukocyte adhesion determined in each mouse was then used to generate a group mean.

### 2.4. Western Blot Analysis

We obtained peritoneal cells from mice to confirm SIRT2 expression. WT, SIRT2KO, and SIRT2KI mice (*n* = 8 each) were injected with sodium thioglycolate medium intraperitoneally, as per literature [25]. All mice developed ascites within four days. On day, four mice were euthanized using isoflurane anesthesia and cervical dislocation (secondary method), and peritoneal lavage was performed to isolate peritoneal macrophages. Western blotting was completed as previously described [18]. The membrane was incubated with antibodies against SIRT-2 (Santa Cruz Biotechnology, sc-20966), GAPDH (Invitrogen, #AM4300), or CPA (Millipore, #07313) overnight followed by the incubation with Alexa Fluor 680-conjugated secondary antibodies (827-10080 or 827-10081; LI-COR Biosciences, Lincoln, NE), and signal was developed with a Li-COR Odyssey Infrared Imager system (Li-COR Biosciences).

### 2.5. Immunohistochemistry

Small intestinal tissue from CLP and sham surgery from all three strains (WT, SIRT2KO, and SIRT2KI) of mice was harvested, and fixed frozen sections were stained using E-selectin and ICAM-1 (BD Biosciences, San Jose, CA, USA) and Von Willebrand factor (VWF, Abcam, Inc., Cambridge, MA, USA). Cy™3-conjugated labeled secondary antibodies for E-selectin, ICAM-1, and FITC-conjugated secondary antibody for VWF were purchased from Jackson ImmunoResearch Laboratories, Inc. (West Grove, PA, USA). The IHC techniques and virtual image captures were as described previously [22, 23].

### 2.6. Experimental Protocol for Sepsis Mice

Mice were subjected to sepsis/sham surgery under isoflurane anesthesia. In IVM experiments, mice with ketamine+xylazine anesthesia underwent carotid/jugular cannulation, mean arterial blood pressure (MAP) measurement via carotid catheter followed by study of leukocyte and platelet adhesion at a 6-hour time point after surgery. A separate group of mice were euthanized with cervical dislocation under anesthesia and tissue harvested for immunohistochemistry. In a third group of mice, we observed a 7-day mortality as indicated. Mice were monitored at least two times per day. Mice were diligently followed for humane end points and euthanized per American Veterinary Medical Association guidelines.

### 2.7. Statistics

Leukocyte/platelet adhesion data were analyzed using Tukey-Kramer post hoc analysis (Statview, SAS; Cary, NC, USA). A *p* < 0.05 was designated as significant. Data for survival rates were analyzed using Graph Pad Prism 6.0 (Graph Pad Software, La Jolla, CA, USA). Log rank test was used to compare survival between the groups in the Kaplan-Meier survival curves and a *p* < 0.05 was designated as significant.

## 3. Results

### 3.1. Sirtuin 2 Modulates Cell Adhesion in Sepsis Microcirculation

To study microvascular inflammation, we assessed sepsis-induced leukocyte and platelet adhesion in small intestinal microcirculation of SIRT2KO and SIRT2KI mice compared to those of WT control. There were no significant differences in the mean arterial blood pressure (MAP), body weight, and total blood leukocyte counts between different groups (Table 1).

**Figure 1:**
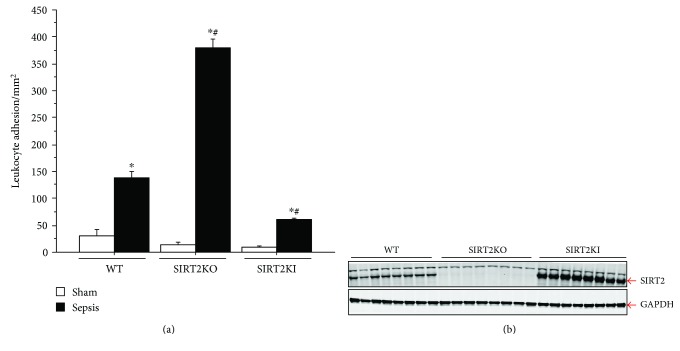
(a) Leukocyte adhesion with SIRT2 modification. Leukocyte adhesion in the small intestinal microcirculation in sepsis groups was significantly increased versus that in the respective sham groups (*n* = 4–6 in each group). Leukocyte adhesion in SIRT2KO sepsis mice was significantly increased versus that in WT sepsis; leukocyte adhesion in SIRT2KI sepsis mice was significantly decreased versus that in WT sepsis mice. Leukocyte adhesion in SIRT2KI sepsis mice was significantly decreased versus that in SIRT2KO sepsis mice. Leukocyte adhesion in WT, SIRT2KO, and SIRT2KI sham mice were not significantly different from each other. ^∗^*p* < 0.05 versus respective sham; ^#^*p* < 0.05 versus WT sepsis using Tukey's post hoc analysis; error bars: sem. (b) shows SIRT2 expression in peritoneal cells from WT, SIRT2KO, and SIRT2KI mice with CLP. As expected, SIRT2 expression in the peritoneal cell in SIRT2KO mice was significantly lower than that in WT while that in SIRT2KI mice was significantly higher compared to that in WT mice.

Figure 1(a) shows that the leukocyte adhesion increased significantly in sepsis versus the respective sham groups in all three strains including WT, SIRT2KO, and SIRT2KI mice. Leukocyte adhesion in the SIRT2KO sepsis group was significantly increased versus that in WT sepsis mice. In contrast, leukocyte adhesion in SIRT2KI sepsis mice was significantly decreased versus that in WT sepsis mice. Lastly, the leukocyte adhesion in SIRT2KI sepsis was significantly attenuated versus that in SIRT2KO mice. Leukocyte adhesion in the WT, SIRT2KO, and SIRT2KI sham groups were not significantly different from each other. We confirmed SIRT2 expression in the peritoneal cells from WT, SIRT2KO, and SIRT2KI. As shown in Figure 1(b), the SIRT2 expression in the peritoneal cells of SIRT2KO mice was lower while that of SIRT2KI mice was higher than that of the WT mice.

**Figure 2:**
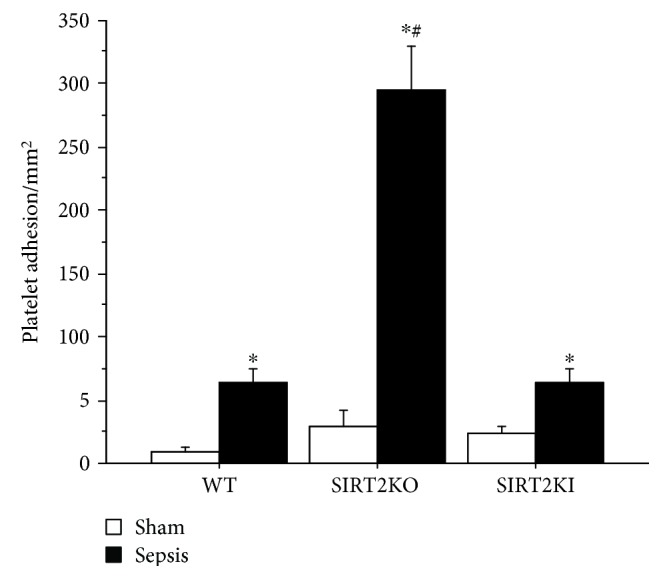
Platelet adhesion with SIRT2 modification. Platelet adhesion in the small intestinal microcirculation in sepsis groups was significantly increased versus that in the respective sham groups (*n* = 4–6 in each group). Platelet adhesion in SIRT2KO sepsis mice was not significantly increased versus that in WT sepsis; platelet adhesion in SIRT2KI sepsis mice was not significantly different versus that in WT sepsis mice. Platelet adhesion in SIRT2KI sepsis mice was significantly decreased versus that in SIRT2KO sepsis mice. Platelet adhesion in WT, SIRT2KO, and SIRT2KI sham mice were not significantly different from each other. ^∗^*p* < 0.05 versus respective sham; ^#^*p* < 0.05 versus WT sepsis using Tukey's post hoc analysis; error bars: sem.

Next, we determined platelet adhesion in WT, SIRT2KO, and SIRT2KI mice with sham and sepsis (Figure 2). Similar to leukocyte adhesion, platelet adhesion in the WT, SIRT2KO, and SIRT2KI sepsis groups significantly increased versus respective sham counterparts. The platelet adhesion in the SIRT2KO sepsis group was significantly increased versus WT sepsis mice. However, unlike leukocyte adhesion, the platelet adhesion in SIRT2KI sepsis mice was not significantly different from that in WT sepsis mice. Lastly, the platelet adhesion in the SIRT2KI sepsis group was significantly lower versus that in SIRT2KO mice. Platelet adhesion in the WT, SIRT2KO, and SIRT2KI sham groups did not differ from each other. Of note, we show total platelet adhesion here; we do not differentiate between platelet-endothelial and leukocyte-platelet-endothelial adhesion in this project.

### 3.2. Sirtuin 2 Regulates Adhesion Molecule Expression in Sepsis Microcirculation

Adhesion molecules are the “early responders” of inflammatory response in microcirculation. To study the mechanism of leukocyte and platelet adhesion in sepsis microcirculation, we defined adhesion molecule expression in small intestinal tissue in the WT, SIRT2KO, and SIRT2KI mice sham and sepsis groups. We focused on NFĸB p65-dependent ICAM-1 and E-selectin, which are dominant endothelial cell-derived modulators of cellular adhesion [26–28].

**Figure 3:**
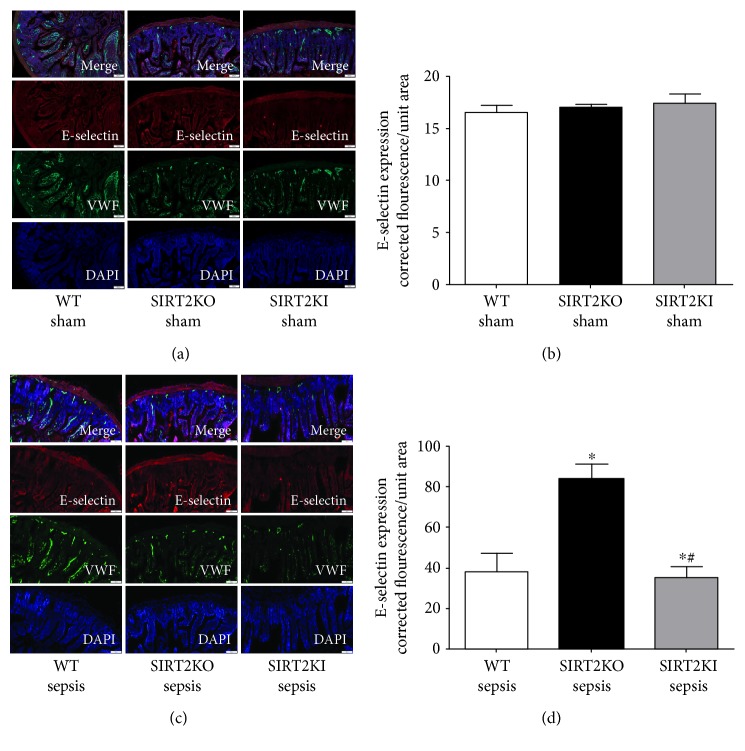
E-selectin expression in WT, SIRT2KO, and SIRT2KI mice. We stained small intestinal tissue for E-selectin expression E-selectin (Cy3: red), Von Willebrand factor (VWF, FITC: green), and nuclear stain (DAPI: blue). (a) shows representative images of E-selectin expression in WT, SIRT2KO, and SIRT2KI mice with sham surgery while (b) shows fluorescence quantification of immunohistochemistry of three different mice in each group. There was no significant difference in E-selectin expression of WT, SIRT2KO, and SIRT2KI mice with sham surgery from each other. As shown in (c) (representative images) and (d) (fluorescence quantification from immunohistochemistry; *n* = 3 in each group), the E-selectin expression increased in SIRT2KO sepsis mice versus that in WT sepsis mice while it was decreased in SIRT2KI sepsis mice versus that in SIRT2KO sepsis mice. ^∗^*p* < 0.05 versus that in WT sepsis group; ^#^*p* < 0.05 versus that in SIRT2KO sepsis group; using Tukey's post hoc analysis; error bars: sem.

As shown in Figures 3(a) and 3(b), there were no significant differences in E-selectin expression between WT, SIRT2KO, and SIRT2KI mice with sham surgery. However, as shown in Figures 3(c) and 3(d), the E-selectin expression in SIRT2KO sepsis mice was significantly higher compared to that in WT sepsis mice while that in SIRT2KI sepsis mice was significantly lower than that in WT sepsis mice. Figures 3(b) and 3(d) show fluorescence quantification from small intestine IHC in each group (*n* = 3) while Figures 3(a) and 3(c) show representative images for E-selectin expression.

**Figure 4:**
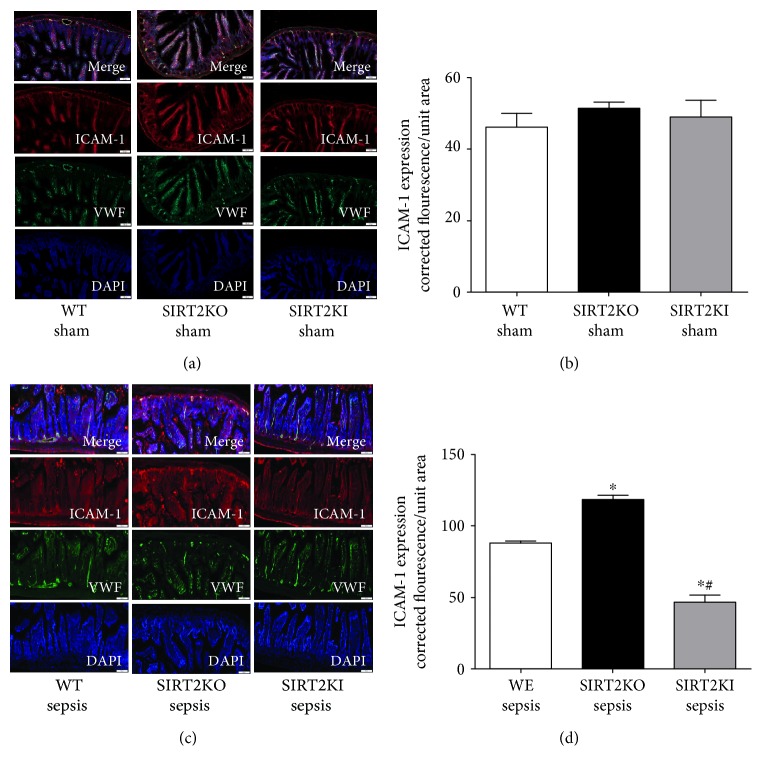
ICAM-1 expression in WT, SIRT2KO, and SIRT2KI mice. We stained small intestinal tissue for ICAM-1 expression. ICAM-1 (Cy3: red), Von Willebrand factor (VWF, FITC: green), and nuclear stain (DAPI: blue). (a) shows representative images of ICAM-1 expression in WT, SIRT2KO, and SIRT2KI mice with sham surgery while (b) shows fluorescence quantification of immunohistochemistry of three different mice in each group. There was no significant difference in ICAM-1 expression of WT, SIRT2KO, and SIRT2KI mice with sham surgery from each other. As shown in (c) (representative images) and (d) (fluorescence quantification from immunohistochemistry; *n* = 3 in each group), the ICAM-1 expression increased in SIRT2KO sepsis mice versus that in WT sepsis mice while it was decreased in SIRT2KI sepsis mice versus that in WT and SIRT2KO sepsis mice. ^∗^*p* < 0.05 versus that in WT sepsis group; ^#^*p* < 0.05 versus that in SIRT2KO sepsis group; using Tukey's post hoc analysis; error bars: sem.

Next, we assessed the ICAM-1 expression in small intestinal tissue in WT, SIRT2KO, and SIRT2KI mice with sepsis. In Figures 4(a) and 4(b), similar to E-selectin expression, we observed no significant difference in ICAM-1 expression between WT, SIRT2KO, and SIRT2KI mice with sham surgery. However, in Figures 4(c) and 4(d), we show increased ICAM-1 expression in SIRT2KO versus WT sepsis and decreased ICAM-1 in SIRT2KI versus SIRT2KO sepsis and WT sepsis. Figures 4(b) and 4(d) show fluorescence quantification from small intestine IHC in each group (*n* = 3) while Figures 4(a) and 4(c) show representative images for ICAM-1 expression. Together, the data support that SIRT2 regulates microvascular inflammation: low SIRT2 increases and high SIRT2 decreases leukocyte/platelet adhesion in sepsis by controlling ICAM-1 and E-selectin expression.

### 3.3. Sirtuin 2 Influences Survival in Sepsis Mice

**Figure 5:**
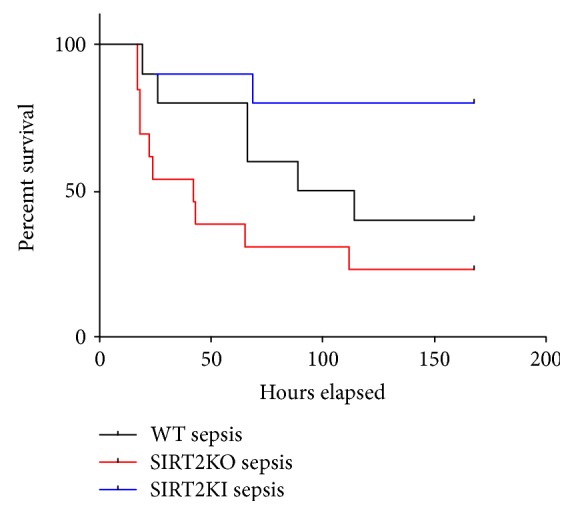
SIRT2 modification impacts sepsis survival. We studied 7-day survival rates in WT, SIRT2KO, and SIRT2KI mice with sepsis. WT sepsis mice showed a 40% 7-day survival rate; SIRT2KO sepsis mice was 10% while SIRT2KI sepsis mice showed an 80% 7-day survival rate.

To test the clinical impact of sepsis-induced inflammation on the whole animal, we determined 7-day survival rates in WT, SIRT2KO, and SIRT2KI mice with sepsis.  Figure 5 depicts a 40% survival rate in WT sepsis mice, 10% in SIRT2KO sepsis, and 80% in SIRT2KI sepsis. This data suggest that the SIRT2 modulates survival in rodent sepsis.

## 4. Discussion

This study identifies a previously unrecognized property of SIRT2 as a regulator of microvascular inflammation. This novel function is via modulating expression of critical adhesion molecules ICAM-1 and E-selectin. Specifically, in an inverse relationship, SIRT2 deficiency is associated with increased cell adhesion and adhesion molecule expressions while SIRT2 overexpression is associated with decreased adhesion and adhesion molecule expressions. The clinical significance of this in mouse sepsis is that sepsis mortality is affected by the SIRT2 modulation as well. These phenotype shifts may represent, at least in part, the known deacetylation-dependent deactivation of NFĸB p65 transcription factor as a master inflammation promoter; we have shown that SIRT2 directly deacetylates NFĸB p65 [18].

This report seems to be in direct contradiction with a previous publication studying that the effect of SIRT2 inhibitor AGK-2 treatment during early sepsis improves survival [29]. There are some key differences between results from that publication and this project. Firstly, while they used a chemical compound to inhibit SIRT2, we used genetic approaches. Off target effects of a chemical compound, namely, AGK-2, cannot be ruled out. Consistent with these results, however, our previous work using a different SIRT2 inhibitor compound during early endotoxemia showed an increased inflammatory response in mouse macrophages. We have shown previously that in fact the AK-7, a SIRT2 inhibitor treatment during the immunosuppressive phase of sepsis in obese mice improved survival. The MVI is sensitive to both local and systemic environmental stimuli such as inflammation; leukocyte adhesion is an essential link to infection outcome [5]. The growing body of evidence suggests a role for platelet activation and adhesion in sepsis related inflammation [30–33]. We have shown previously that the leukocyte and platelet adhesion increase during early sepsis [7, 34, 35]. In this project, we show the effect of SIRT2 modification on platelet adhesion along with leukocyte adhesion.

The selectins are responsible for the rolling and initial capture of leukocytes, while the engagement of leukocyte-*β*2-integrins with endothelial ICAM-1 mediates “firm” adhesion followed by emigration of leukocytes [36]. We previously reported that early sepsis increases adhesion molecule expression in MVI [7, 37]. Moreover, we reported that SIRT1 modulates the E-selectin and ICAM-1 expression during sepsis [22, 23]. This study adds SIRT2 as MVI regulator via E-selectin and ICAM-1 expression. Together, our findings support a role for both SIRT1 and SIRT2 in epigenetically directing inflammation. However, whether there are separate effects of the two SIRTs or redundancies will require further study.

Sirtuins are NAD+ sensors in immune and nonimmune cells. We have shown previously that SIRT2 deficiency in immune cells exaggerates inflammatory response via reacetylation of NFĸB p65 [18]. SIRT2 is abundantly expressed in various tissue beds such as the brain, adipose tissue, liver, muscle, kidney, and pancreas. [15, 38–40]. In the current manuscript, we confirmed that the whole body SIRT2KO and SIRT2KI mice show changes in SIRT2 expression immune cells, namely, peritoneal macrophages as well. SIRT2 is selective for deacetylation of tubulin and contributes to HIF1-α to modulate hypoxia-dependent responses in tumor cells [41]. Clearly, SIRT2 acts in cytosol and in the nucleus during a stress response. It is possible that SIRT2 joins SIRTs1 and 6 in nuclear control of inflammation but may act selectively on cytosolic tubulin. It seems likely that the sirtuin family functions in a cooperative manner for the control of inflammation. How this network is informed of stress is unknown but might involve shifts in redox signaling. Sepsis generates biphasic oxidative stress [42]. Sirtuins are known to activate an antioxidant response via the FOXO transcription family; SIRT2 modulates FOXO3a during oxidative stress [40]. Although we have not pursued this line of investigation, it is possible that SIRT2 modulates a microvascular inflammatory response via this pathway. SIRT1 and 2, in endothelial cells, might exhibit nonoverlapping activities [43]. More studies are required to separate cooperation from distinct pathway or protein contributions.

In conclusion, the study, for the first time to our knowledge, supports a critical role for SIRT2 in regulating MVI responses and in influencing sepsis mortality in mice. This finding may open the way to novel therapies for adjusting MVI pathobiology during the inflammatory response.

## Figures and Tables

**Table 1:** Mean arterial blood pressure (MAP) and body weight in different groups of mice: the MAP, body weight, and total leukocyte count of different groups (mean ± sem; *n* = 5 per group) of mice studied did not show a significant difference from each other using Tukey's post hoc analysis.

	MAP (mmHg)	Body weight (gm)	Total leukocyte count (per mm^3^)
WT sham	58 ± 4.3	23 ± 1.2	6896 ± 1221
WT sepsis	64 ± 2.3	25 ± 1	4542 ± 1855
SIRT2KO sham	60 ± 5.7	25 ± 1	3667 ± 654
SIRT2KO sepsis	63 ± 4.8	26 ± 0.5	3484 ± 445
SIRT2KI sham	59 ± 3.2	23 ± 1.2	3542 ± 167
SIRT2KI sepsis	57 ± 2.0	21 ± 1.5	3229 ± 127

## Abbreviations

CLP: Cecal ligation and puncture
ICAM: Intracellular adhesion molecule
MVI: Microvascular interface
NAD: Nicotinamide dinucleotide
NFĸB: Nuclear factor kappa B
SIRT: Sirtuin
SIRT1: Sirtuin 1
SIRT2: Sirtuin 2
SIRT2KI: Sirtuin 2 overexpressing
SIRT2KO: Sirtuin 2 knockout
WT: Wild type (lean).

## References

[1] Torio C. M., Andrews R. M. (2006). National inpatient hospital costs: the most expensive conditions by payer, 2011: statistical brief #160. *Healthcare Cost and Utilization Project (HCUP) Statistical Briefs*.

[2] Torio C. M., Moore B. J. (2006). National inpatient hospital costs: the most expensive conditions by payer, 2013: statistical brief #204. *Healthcare Cost and Utilization Project (HCUP) Statistical Briefs*.

[3] Williams S. C. (2012). After Xigris, researchers look to new targets to combat sepsis. *Nature Medicine*.

[4] Marshall J. C., Cook D. J., Christou N. V., Bernard G. R., Sprung C. L., Sibbald W. J. (1995). Multiple organ dysfunction score: a reliable descriptor of a complex clinical outcome. *Critical Care Medicine*.

[5] Jung U., Norman K. E., Scharffetter-Kochanek K., Beaudet A. L., Ley K. (1998). Transit time of leukocytes rolling through venules controls cytokine-induced inflammatory cell recruitment in vivo. *The Journal of Clinical Investigation*.

[6] Singer G., Stokes K. Y., Terao S., Granger D. N. (2009). Sepsis-induced intestinal microvascular and inflammatory responses in obese mice. *Shock*.

[7] Vachharajani V., Russell J. M., Scott K. L. (2005). Obesity exacerbates sepsis-induced inflammation and microvascular dysfunction in mouse brain. *Microcirculation*.

[8] Li X., Zhang S., Blander G., Tse J. G., Krieger M., Guarente L. (2007). SIRT1 deacetylates and positively regulates the nuclear receptor LXR. *Molecular Cell*.

[9] Lombard D. B., Schwer B., Alt F. W., Mostoslavsky R. (2008). SIRT6 in DNA repair, metabolism and ageing. *Journal of Internal Medicine*.

[10] Vachharajani V., Liu T., McCall C. E. (2014). Epigenetic coordination of acute systemic inflammation: potential therapeutic targets. *Expert Review of Clinical Immunology*.

[11] Nakagawa T., Guarente L. (2011). Sirtuins at a glance. *Journal of Cell Science*.

[12] Liu T. F., Yoza B. K., El Gazzar M., Vachharajani V. T., McCall C. E. (2011). NAD+−dependent SIRT1 deacetylase participates in epigenetic reprogramming during endotoxin tolerance. *The Journal of Biological Chemistry*.

[13] Liu T. F., Vachharajani V., Millet P., Bharadwaj M. S., Molina A. J., McCall C. E. (2015). Sequential actions of SIRT1-RELB-SIRT3 coordinate nuclear-mitochondrial communication during immunometabolic adaptation to acute inflammation and sepsis. *The Journal of Biological Chemistry*.

[14] Liu T. F., Vachharajani V. T., Yoza B. K., McCall C. E. (2012). NAD+−dependent sirtuin 1 and 6 proteins coordinate a switch from glucose to fatty acid oxidation during the acute inflammatory response. *The Journal of Biological Chemistry*.

[15] Gomes P., Outeiro T. F., Cavadas C. (2015). Emerging role of sirtuin 2 in the regulation of mammalian metabolism. *Trends in Pharmacological Sciences*.

[16] North B. J., Verdin E. (2007). Mitotic regulation of SIRT2 by cyclin-dependent kinase 1-dependent phosphorylation. *The Journal of Biological Chemistry*.

[17] Inoue T., Hiratsuka M., Osaki M. (2007). SIRT2, a tubulin deacetylase, acts to block the entry to chromosome condensation in response to mitotic stress. *Oncogene*.

[18] Wang X., Buechler N. L., Martin A. (2016). Sirtuin-2 regulates sepsis inflammation in ob/ob mice. *PloS One*.

[19] Lin J., Sun B., Jiang C., Hong H., Zheng Y. (2013). Sirt2 suppresses inflammatory responses in collagen-induced arthritis. *Biochemical and Biophysical Research Communications*.

[20] Yuan F., Xu Z. M., Lu L. Y. (2016). SIRT2 inhibition exacerbates neuroinflammation and blood-brain barrier disruption in experimental traumatic brain injury by enhancing NF-kappaB p65 acetylation and activation. *Journal of Neurochemistry*.

[21] North B. J., Rosenberg M. A., Jeganathan K. B. (2014). SIRT2 induces the checkpoint kinase BubR1 to increase lifespan. *The EMBO Journal*.

[22] Vachharajani V. T., Liu T., Brown C. M. (2014). SIRT1 inhibition during the hypoinflammatory phenotype of sepsis enhances immunity and improves outcome. *Journal of Leukocyte Biology*.

[23] Wang X., Buechler N. L., Yoza B. K., McCall C. E., Vachharajani V. T. (2015). Resveratrol attenuates microvascular inflammation in sepsis via SIRT-1-induced modulation of adhesion molecules in ob/ob mice. *Obesity*.

[24] Tailor A., Granger D. N. (2003). Hypercholesterolemia promotes P-selectin-dependent platelet-endothelial cell adhesion in postcapillary venules. *Arteriosclerosis, Thrombosis, and Vascular Biology*.

[25] Wang X., Cao Q., Yu L., Shi H., Xue B., Shi H. (2016). Epigenetic regulation of macrophage polarization and inflammation by DNA methylation in obesity. *JCI Insight*.

[26] Dieterich L. C., Huang H., Massena S., Golenhofen N., Phillipson M., Dimberg A. (2013). alphaB-crystallin/HspB5 regulates endothelial-leukocyte interactions by enhancing NF-kappaB-induced up-regulation of adhesion molecules ICAM-1, VCAM-1 and E-selectin. *Angiogenesis*.

[27] Jiang Y., Jiang L. L., Maimaitirexiati X. M., Zhang Y., Wu L. (2015). Irbesartan attenuates TNF-alpha-induced ICAM-1, VCAM-1, and E-selectin expression through suppression of NF-kappaB pathway in HUVECs. *European Review for Medical and Pharmacological Sciences*.

[28] Kim I., Moon S. O., Kim S. H., Kim H. J., Koh Y. S., Koh G. Y. (2001). Vascular endothelial growth factor expression of intercellular adhesion molecule 1 (ICAM-1), vascular cell adhesion molecule 1 (VCAM-1), and E-selectin through nuclear factor-kappa B activation in endothelial cells. *The Journal of Biological Chemistry*.

[29] Zhao T., Alam H. B., Liu B. (2015). Selective inhibition of SIRT2 improves outcomes in a lethal septic model. *Current Molecular Medicine*.

[30] Kiefmann R., Heckel K., Schenkat S., Dorger M., Wesierska-Gadek J., Goetz A. E. (2004). Platelet-endothelial cell interaction in pulmonary micro-circulation: the role of PARS. *Thrombosis and Haemostasis*.

[31] Kupper S., Mees S. T., Gassmann P., Brodde M. F., Kehrel B., Haier J. (2007). Hydroxyethyl starch normalizes platelet and leukocyte adhesion within pulmonary microcirculation during LPS-induced endotoxemia. *Shock*.

[32] Morganti R. P., Marcondes S., Baldasso P. A., Marangoni S., De Nucci G., Antunes E. (2008). Inhibitory effects of staphylococcal enterotoxin type B on human platelet adhesion in vitro. *Platelets*.

[33] Sun W., Li F. S., Zhang Y. H., Wang X. P., Wang C. R. (2015). Association of susceptibility to septic shock with platelet endothelial cell adhesion molecule-1 gene Leu125Val polymorphism and serum sPECAM-1 levels in sepsis patients. *International Journal of Clinical and Experimental Medicine*.

[34] Vachharajani V., Cunningham C., Yoza B., Carson J., Vachharajani T. J., McCall C. (2012). Adiponectin-deficiency exaggerates sepsis-induced microvascular dysfunction in the mouse brain. *Obesity*.

[35] Vachharajani V., Vital S., Russell J., Scott L. K., Granger D. N. (2006). Glucocorticoids inhibit the cerebral microvascular dysfunction associated with sepsis in obese mice. *Microcirculation*.

[36] Granger D. N. (1997). Cell adhesion and migration. II. Leukocyte-endothelial cell adhesion in the digestive system. *The American Journal of Physiology*.

[37] Vachharajani V., Wang S. W., Mishra N., El Gazzar M., Yoza B., McCall C. (2010). Curcumin modulates leukocyte and platelet adhesion in murine sepsis. *Microcirculation*.

[38] Jing E., Gesta S., Kahn C. R. (2007). SIRT2 regulates adipocyte differentiation through FoxO1 acetylation/deacetylation. *Cell Metabolism*.

[39] Kim H. S., Vassilopoulos A., Wang R. H. (2011). SIRT2 maintains genome integrity and suppresses tumorigenesis through regulating APC/C activity. *Cancer Cell*.

[40] Wang F., Nguyen M., Qin F. X., Tong Q. (2007). SIRT2 deacetylates FOXO3a in response to oxidative stress and caloric restriction. *Aging Cell*.

[41] Seo K. S., Park J. H., Heo J. Y. (2015). SIRT2 regulates tumour hypoxia response by promoting HIF-1alpha hydroxylation. *Oncogene*.

[42] Liu Y. C., Chang A. Y., Tsai Y. C., Chan J. Y. (2009). Differential protection against oxidative stress and nitric oxide overproduction in cardiovascular and pulmonary systems by propofol during endotoxemia. *Journal of Biomedical Science*.

[43] Liu J., Wu X., Wang X. (2013). Global gene expression profiling reveals functional importance of SIRT2 in endothelial cells under oxidative stress. *International Journal of Molecular Sciences*.

